# Coupling high-throughput genetics with phylogenetic information reveals an epistatic interaction on the influenza A virus M segment

**DOI:** 10.1186/s12864-015-2358-7

**Published:** 2016-01-12

**Authors:** Nicholas C. Wu, Yushen Du, Shuai Le, Arthur P. Young, Tian-Hao Zhang, Yuanyuan Wang, Jian Zhou, Janice M. Yoshizawa, Ling Dong, Xinmin Li, Ting-Ting Wu, Ren Sun

**Affiliations:** Department of Molecular and Medical Pharmacology, David Geffen School of Medicine, University of California, Los Angeles, 90095 CA USA; Molecular Biology InstituteUniversity of California, Los Angeles, 90095 CA USA; Department of Integrative Structural and Computational Biology, The Scripps Research Institute, La Jolla, 92037 CA USA; Department of Microbiology, Third Military Medical University, Chongqing, 400038 China; Department of Pathology and Laboratory Medicine, David Geffen School of Medicine, University of California, Los Angeles, 90095 CA USA

**Keywords:** Mutagenesis, Fitness profiling, Natural sequence variation, Coevolution analysis, Compensatory mutation

## Abstract

**Background:**

Epistasis is one of the central themes in viral evolution due to its importance in drug resistance, immune escape, and interspecies transmission. However, there is a lack of experimental approach to systematically probe for epistatic residues.

**Results:**

By utilizing the information from natural occurring sequences and high-throughput genetics, this study established a novel strategy to identify epistatic residues. The rationale is that a substitution that is deleterious in one strain may be prevalent in nature due to the presence of a naturally occurring compensatory substitution. Here, high-throughput genetics was applied to influenza A virus M segment to systematically identify deleterious substitutions. Comparison with natural sequence variation showed that a deleterious substitution M1 Q214H was prevalent in circulating strains. A coevolution analysis was then performed and indicated that M1 residues 121, 207, 209, and 214 naturally coevolved as a group. Subsequently, we experimentally validated that M1 A209T was a compensatory substitution for M1 Q214H.

**Conclusions:**

This work provided a proof-of-concept to identify epistatic residues by coupling high-throughput genetics with phylogenetic information. In particular, we were able to identify an epistatic interaction between M1 substitutions A209T and Q214H. This analytic strategy can potentially be adapted to study any protein of interest, provided that the information on natural sequence variants is available.

**Electronic supplementary material:**

The online version of this article (doi:10.1186/s12864-015-2358-7) contains supplementary material, which is available to authorized users.

## Background

Epistasis is a critical factor in viral evolution [1, 2], in which the phenotypic effect of a given mutation varies under different genetic backgrounds. The importance of epistasis has been demonstrated in drug resistance [3–5], immune escape [6, 7], and cross-species adaptation [8]. Therefore, identification of pairwise epistatic interaction offers valuable information to understand the functional basis of viral evolution in nature.

Several virus sequence databases are publicly available [9–11], which permit interrogation of evolutionary pathways in nature and allow approximation of the chronological order of mutation accumulation [6, 12]. Numerous computational algorithms and analytical tools have been developed to identify molecular interactions based on coevolving residues (reviewed in [13]). Such phylogenetic information may lead to the identification of epistatic interactions [5, 12]. However, coevolving mutations may be attributed to genetic drift and hitchhiking, which can be pervasive in evolution [14–16], rather than epistatic interactions. Subsequently, many different combinations of mutations have to be individually constructed and analyzed to discern epistatic residues. It becomes inefficient to probe for epistatic interaction based on coevolutionary analysis without any prior knowledge of the mutational fitness effect.

Recently, high-throughput genetics becomes a popular strategy to profile the fitness effects of a large number of mutations in parallel [17]. The basis of high-throughput genetics is to generate a panel of mutations using high-throughput mutagenesis, and to use deep sequencing to monitor the occurrence frequency of individual mutations when selection is imposed. The change of frequency of each mutation can then be translated into a fitness effect. High-throughput genetics opens up the opportunities to identify critical residues in the protein of interest under any given selection condition. A medically important application is to systematically investigate the effects of mutations in a virus gene or genome [18–23]. It has been shown that high-throughput genetics facilitates the identification of drug resistance substitutions [18], anti-interferon residues [24], and understanding of the evolution of circulating viral strains [20].

High-throughput genetics is often applied to examine mutational fitness effect under only one genetic background of a virus species in one study. However, due to epistasis, a given mutation may have a very different fitness effect among different genetic backgrounds in nature [12, 25]. Therefore, it is not surprising that some mutations with a low replication fitness in a laboratory strain can be prevalent in nature. Indeed, such observation has been made in a high-throughput genetics study of the influenza A virus hemagglutinin protein [21]. However, it is not always straightforward to identify the genetic determinant underlying the epistatic effect.

Matrix (M) segment is of the influenza A virus encodes two proteins, namely M1 and M2. M1 is the matrix protein that forms a protein coat inside the viral envelop. It plays an important role in virus assembly and budding [26, 27]. M2 is a proton-selective ion channel that facilitates the uncoating of virions in the infected cells [28]. In addition, both M1 and M2 are critical determinants in the morphology of the viral particles [29]. While M2 is a major target for the development of anti-influenza drug [30], resistance mutations can rapidly emerge without any cost on viral replication fitness [31, 32]. On the other hand, being a highly conserved protein, M1 is an effective antigen to drive heterosubtypic protection through T-cell immunity [33, 34]. In fact, M1 has been used as a target for the development of T-cell-based vaccine against influenza virus [35]. Due to the biomedical significance of the M segment of influenza A virus, it is important to comprehend the fitness consequences of individual mutations and epistatic interactions among mutations in M1 and M2.

In this study, we described an approach to identify pairwise epistatic interaction by coupling high-throughput genetics with phylogenetic information. Using high-throughput genetics, we were able to systematically identify deleterious substitutions in the M segment of influenza virus A/WSN/33. Three substitutions that were classified as deleterious were prevalence in the circulating strains. A phylogenetic analysis on the circulating strains was then performed to examine whether those substitutions of interest were coevolving with other residues. These analyses led us to identify and experimentally validate the epistatic interaction between A209T and Q214H, in which A209T was able to compensate the deleterious effect of Q214H. Interestingly, both substitutions were prevalent in the 2009 pandemic swine influenza virus strains, but not in the seasonal influenza virus strains. This study demonstrates the power of combining high-throughput genetics and phylogenetic information to identify epistatic residues.

## Results

### Methodology overview and experimental design

**Fig. 1:**
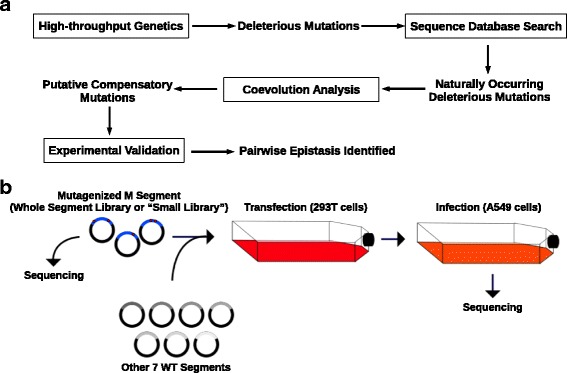
Methodology overview and experimental design. **a** The proposed workflow for identifying pairwise epistatic interaction is shown. Key methodologies are boxed. **b** The experimental scheme is shown. Briefly, 293T cells (represented by the red flask) were transfected with the randomly mutagenized M segment (DNA library) and the other seven WT segments to generate the viral mutant library. This viral mutant library was used to infect A549 cells (represented by the orange flask) for 24-hour to generate the post-infection library. The DNA library and the post-infection library were subjected to deep sequencing

The goal of this study was to develop a methodology to systematically identify pairwise epistatic interaction, more specifically between deleterious mutations and compensatory mutations. We proposed to couple high-throughput genetics with phylogenetic information to achieve such purpose (Fig. 1a). First, high-throughput genetics could be utilized to identify deleterious mutations. Second, sequence database was explored to determine whether any of those deleterious mutations could be observed in naturally occurring sequences. Third, if a deleterious mutation could be observed in naturally occurring sequences, a coevolution analysis would be performed to identify potential compensatory mutations. Such putative epistatic interaction would then need to be confirmed experimentally. In this study, we provided a proof-of-concept using the M segment of influenza virus.

High-throughput genetics has been applied to study 7 out of 8 segments of influenza A virus genome, which include PB2 segment [36], PB1 segment [36], PA segment [23, 36], HA segment [19, 21], NP segment [20], NA segment [37], and NS segment [24]. In this study, the M segment was analyzed by high-throughput genetics. Two different mutant libraries were built, namely the whole segment mutant library and “small libraries”. For the whole segment mutant library, the entire M segment was subjected to mutagenesis. In contrast, for each “small library”, only a 240-bp region was mutagenized. ∼94 % of the nucleotide position of the M segment was covered by the whole segment mutant library, or by four different “small libraries”.

Each mutant library was transfected in 293T cells and the resultant viral mutant library was used to infect A549 cells for 24 hours (Fig. 1b). Both the plasmid mutant library and the post-infection mutant library were subjected to deep sequencing. Biological replicates were obtained by independent transfection and infection. We have included two biological replicates for the whole segment mutant library (replicate 1 and 2) and three biological replicates for each of the “small libraries” (replicate 3 to 5). The sequencing coverage for each sample is shown in Table 1.

**Table 1:** Sequencing coverage

Replicate	Library type	Average	Minimum	Maximum
		coverage	coverage	coverage
DNA input	Whole segment	157,846	82,998	189,371
DNA input	Small libraries	54,850	44,297	105,183
1	Whole segment	242,390	158,210	276,850
2	Whole segment	43,286	11,451	131,578
3	Small libraries	59,694	30,003	113,619
4	Small libraries	50,758	29,606	91,134
5	Small libraries	63,659	18,201	104,731

For those replicates with the library type indicated as “Whole Segment”, the coverage represents the number of error-corrected reads [19]. For those replicates with the library type indicated as “Small Libraries”, the coverage represents the number of sequencing reads

### Estimation of fitness effect for individual point mutations

**Fig. 2:**
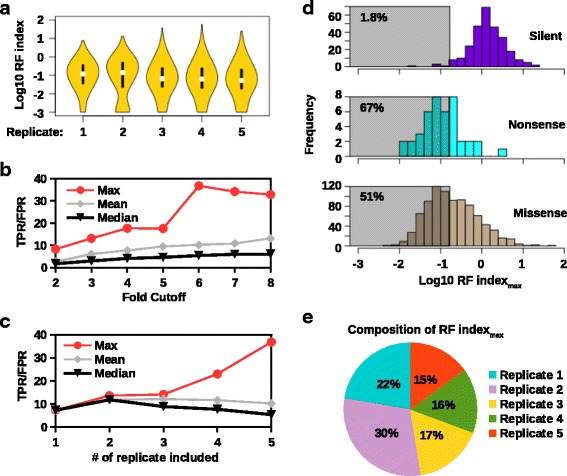
Systematic identification of deleterious mutations. **a** The distributions of RF index in different replicates are shown as violin plots. The white circle at the center represents the median and the black box represents the interquartile range. RF index of < 0.001 was set to 0.001 here for visualization purpose. **b** The ratio of true positive rate (TPR) to false positive rate (FPR) for classifying deleterious mutations was evaluated across different cutoffs. All five replicates were used in this analysis. **c** The ratio of TPR to FPR for classifying deleterious mutations was computed as the number of replicate being used to generate RF index increases. **b** and **c** RF index _*max*_, RF index _*mean*_, and RF index _*median*_ were analyzed. The red line represents RF index _*max*_. The grey line represents RF index _*mean*_. The black line represents RF index _*median*_. **d** The distributions of RF index _*max*_ for silent mutations, nonsense mutations, and missense mutations are shown as histograms. The shaded area represents the range of RF index _*max*_ where mutations were identified as deleterious. The percentage of mutations being identified as deleterious is indicated. **e** The composition of RF index _*max*_ is shown as a pie chart

Relative fitness index (RF index), which was computed as the enrichment ratio of the relative occurrence frequency _*post*−*infection*_ to the relative occurrence frequency _*plasmidmutantlibrary*_ [19, 23], was used as a proxy for the fitness effect of individual point mutations. For each point mutation, five independent RF indices were obtained from five replicates. Although the distribution of RF index in different replicates are similar (Fig. 2a), the Spearman’s rank correlation coefficient between RF indices for individual mutations across different replicates is only moderate, ranging from 0.53 to 0.67 (Table 2). The lack of a strong correlation can be attributed to the bottleneck of genetic diversity in the transfection step as described in other high-throughput genetic studies using the influenza reverse genetic system [20, 21]. This bottleneck would result in a limited number of virus mutations being reconstituted from the plasmid mutant library. In other words, even though some mutations were present in the plasmid mutant library, they may not be reconstituted into the viral mutant library due to the bottleneck in the transfection step. Those mutations that were not reconstituted into the viral mutant library may not be deleterious, but would be identified as deleterious due to their absence in the post-infection pool. This bottleneck can be viewed as an incomplete sampling process of the plasmid mutant library. Our recent study suggested that the bottleneck effect could be relieved by scaling up the transfection by using more DNA plasmid and more 293T cells [23].

**Table 2:** Correlations of fitness profile across replicates

Correlation	Replicate 1	Replicate 2	Replicate 3	Replicate 4	Replicate 5
Replicate 1	1.00	0.67	0.61	0.56	0.53
Replicate 2	0.67	1.00	0.59	0.57	0.54
Replicate 3	0.61	0.59	1.00	0.56	0.58
Replicate 4	0.56	0.57	0.56	1.00	0.55
Replicate 5	0.53	0.54	0.58	0.55	1.00

The Spearman’s rank correlation coefficient between RF indices for individual mutations across different replicates are shown

### Systematic identification of deleterious mutations

The ratio of true positive rate (TPR) to false positive rate (FPR) was used to evaluate the statistical confidence in the identification of deleterious mutations. In the following, this ratio would be abbreviated as TPR/FPR. TPR was computed as the fraction of nonsense mutations, which were expected to be phenotypically lethal, being identified as deleterious. FPR was computed as the fraction of silent mutations, which were expected to be phenotypically neutral, being identified as deleterious. TPR/FPR could be regarded as a measure of signal-to-noise ratio for the identification of deleterious mutations. A larger value of TPR/FPR represented a higher confidence in the identification of deleterious mutations. We acknowledged that FPR may be slightly overestimated because it is known that some silent mutations may impose a fitness cost.

We tested different cutoffs for RF index for the identification of deleterious mutations (Fig. 2b). To compile the five RF indices from five replicates (two whole segment mutant library replicates and three “small libraries” replicates) into one single RF index for a given mutation, we proposed three different measures: 1) the highest value among the five RF indices from those five replicates (RF index _*max*_) was used, 2) the average value of the five RF indices from those five replicates (RF index _*mean*_) was used, and 3) the median value of the five RF indices from those five replicates (RF index _*median*_). A mutation would be identified as deleterious when its RF index was less than the indicated cutoff. Here, all three measures of RF index (RF index _*max*_, RF index _*mean*_, and RF index _*median*_) were tested against seven different cutoffs, ranging from 2-fold to 8-fold decreased in relative occurrence frequency from plasmid mutant library to post-infection library (equivalent to an RF index of 1/2=0.5 to 1/8=0.125). The TPR/FPR of both RF index _*mean*_ and RF index _*median*_ were lowered than that of RF index _*max*_ across all tested cutoff. This indicates that RF index _*max*_ would give the highest confidence among all three measures of RF index in identifying deleterious mutations. For RF index _*max*_, TPR/FPR was peaked at 36.8 with a cutoff of 6-fold decreased in relative occurrence frequency (RF index _*max*_=1/6≈ 0.167). In other words, there would be a 36.8-fold enrichment of deleterious mutations over non-deleterious mutations using a 6-fold cutoff for RF index _*max*_.

We further tested the impact of including different number of replicates on the confidence in the identification of deleterious mutations. A monotonic increase in TPR/FPR was observed as more replicates were included in the calculation of RF index _*max*_, indicating the benefit of having more replicates in the identification of deleterious mutations (Fig. 2c). In contrast, an increase in the number of replicate did not increase TPR/FPR for both RF index _*mean*_ and RF index _*median*_. Again, this result shows the advantage of using RF index _*max*_ instead of RF index _*mean*_ or RF index _*median*_ in the identification of deleterious mutations. Subsequently, a 6-fold cutoff for RF index _*max*_ was employed for the rest of this study, in which 1.8 % of silent mutations, 67 % of nonsense mutations, and 51 % of missense mutations were identified as deleterious (Fig. 2d).

We postulated that due to the presence of the bottleneck effect in the transfection step, the usage of RF index _*max*_ was more efficient than RF index _*mean*_ and RF index _*median*_ in the identification of deleterious mutations. As mentioned above, bottleneck effect in the transfection step would lead to a neutral mutation being identified as a deleterious mutation. However, since the bottleneck was independent in each replicate, the probability for a neutral mutation being identified as neutral in at least one replicate increased as the number of replicates increased. Whereas a deleterious mutation should be identified as deleterious regardless of the number of replicates. Therefore, the power of using RF index _*max*_ to distinguish deleterious mutations versus non-deleterious mutations would increase as the number of replicates increased. In contrast, as our results suggest, the power of using RF index _*mean*_ or RF index _*median*_ to distinguish deleterious mutations versus non-deleterious mutations would not benefit from an increasing number of replicates. Since the goal here was to confidently identify deleterious mutations using the data from five replicates, the usage RF index _*max*_, was more suitable than RF index _*mean*_ or RF index _*median*_.

The composition of the RF index _*max*_ was examined (Fig. 2e). Replicate 2 contributed the most to the RF index _*max*_, in which 30 % of the RF index _*max*_ came from replicate 2. Replicate 5 contributed the least to the RF index _*max*_, in which 15 % of the RF index _*max*_ came from replicate 5. This variation in contribution to RF index _*max*_ was likely due to different degrees of bottleneck effect in each replicate.

### Validation and functional relevance of the high-throughput genetics result

**Fig. 3:**
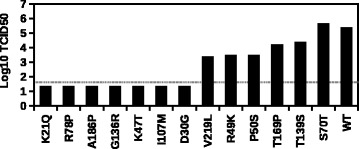
Validation of the profiling result by virus rescue experiment. Based on the profiling result, 13 randomly selected deleterious substitutions (RF index _*max*_< 0.167) were reconstructed and analyzed by virus rescue experiment. The TCID_50_ measured from the virus rescue experiment is shown. The grey dashed line represents the lower detection limit

To experimentally confirmed the reliability of our dataset, we randomly selected and individually reconstructed 13 substitutions on M1 that were identified as deleterious (RF index _*max*_< 0.167). A virus rescue experiment was performed to assess the fitness effect of these substitutions. Seven substitutions (K21Q, R78P, A186P, G136R, K47T, I107M, and D30G) had undetectable viral titer, three substitutions (V219L, R49K, and P50S) had two-log drop in viral titer as compared to wild-type (WT), two substitutions (T169P and T139S) had one-log drop in viral titer as compared to WT, and only one substitution (S70T) had WT-like viral titer (Fig. 3). Overall, 12 out of 13 substitutions displayed a deficiency in viral replication. Note that, deficiency in viral replication was defined by at least 10-fold decrease in viral titer in the rescue experiment, which was a reasonable cutoff as indicated by a large-scale mutational analysis of influenza A virus nucleoprotein [38]. This experiment validated our approach in identifying deleterious substitutions.

**Fig. 4:**
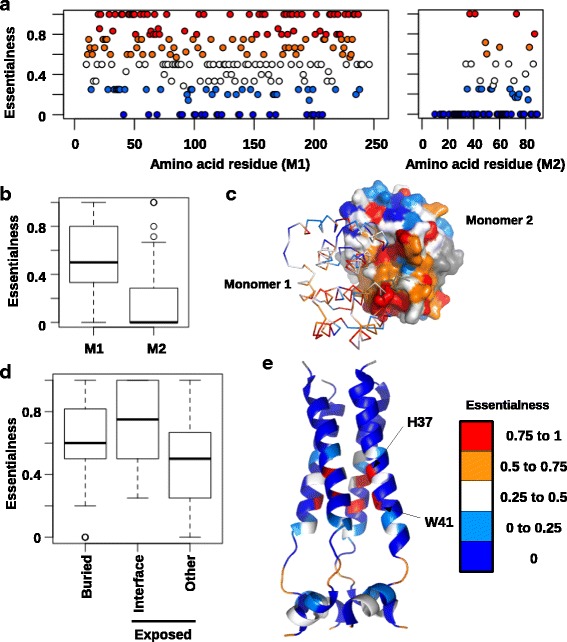
Functional relevance of the profiling result. **a** At each amino acid residue, essentialness represents the fraction of profiled substitutions being deleterious. The essentialness for those residue with ≥ 2 substitutions being profiled is shown. Each data point is colored according to the value of essentialness: essentialness = 0 (blue), 0 < essentialness ≤ 0.25 (marine), 0.25 < essentialness ≤ 0.5 (white), 0.5 < essentialness ≤ 0.75 (orange), 0.75 < essentialness ≤ 1 (red). **b** The distributions of essentialness for individual residues on M1 and M2 are shown as boxplots. **c** The essentialness is projected on the structure of homodimer of M1 N-terminal domain (PDB: 1EA3) [39]. Residues are color-coded as that of panel **a**. Those residues with < 2 substitutions being profiled is colored in grey. **d** Individual residues on M1 N-terminal domain were categorized into buried residues, surface-exposed residues at the homodimer interface, and other surface-exposed residues. The distributions of essentialness for these three categories are shown as boxplots. **e** The essentialness is projected on the structure of homotetramer of M2 ion channel (PDB: 2RLF) [72]. Residues are color coded according to that of panel **a**. Those residues with < 2 substitutions being profiled is colored in grey

We aimed to further confirm the functional relevance of our the high-throughput genetics data by analyzing the essentialness of individual residues. For each amino acid residue, essentialness was computed as the fraction of profiled substitutions being deleterious (Fig. 4a-b). In general, residues on M1 protein (mean essentialness =0.55, median essentialness =0.5) were more essential, hence less mutable, than residues on M2 protein (mean essentialness =0.19, median essentialness =0) (*P* = 1.7×10^−15^, Wilcoxon rank-sum test). Projecting the essentialness on the structure of M1 revealed the non-mutability of the M1-M1 interface (Fig. 4c), which was important for the oligomerization of M1 [39] and was required for matrix layer formation during assembly and budding [40]. A quantitative analysis was performed to compare the essentialness of buried residues, residues at the dimeric interface, and other surfaced-exposed residues (see “Methods” section for the classification scheme). The essentialness for residues at the dimeric interface is significantly higher than that of other surface-exposed residues (*P* = 0.04, Wilcoxon rank-sum test) (Fig. 4d). In fact, the essentialness of buried residues is also significantly higher than that of other surface-exposed residues (*P*=0.04, Wilcoxon rank-sum test) but has no significant difference with that of residues at the dimeric interface (*P* = 0.33, Wilcoxon rank-sum test). This analysis confirmed the essentialness of the M1-M1 interface.

For M2, only two highly essential residues, H37 and W41, were observed on the structure (Fig. 4e). These two residues are absolutely required for the ion channel function [41, 42], in which H37 acts as a selectivity filter [43, 44] and W41 acts as a channel gate [45, 46]. Overall, these analyses demonstrate the functional relevance of our high-throughput genetics result.

### Discrepancy between natural sequence variation and fitness profiling data

**Fig. 5:**
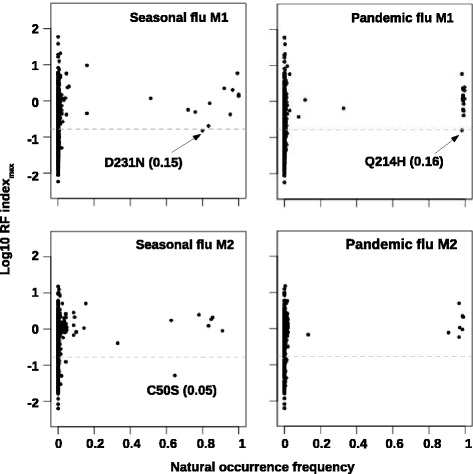
Comparison between natural variation and profiling result. The relationship between RF index _*max*_ for individual amino acid substitutions and the occurrence frequency in natural circulating strains is shown. This comparison was performed on both M1 and M2 proteins with seasonal influenza virus strains (Seasonal flu) and 2009 pandemic swine influenza virus strains (Pandemic flu) being analyzed independently. The grey dashed line represents the cutoff for classifying mutations as deleterious

**Fig. 6:**
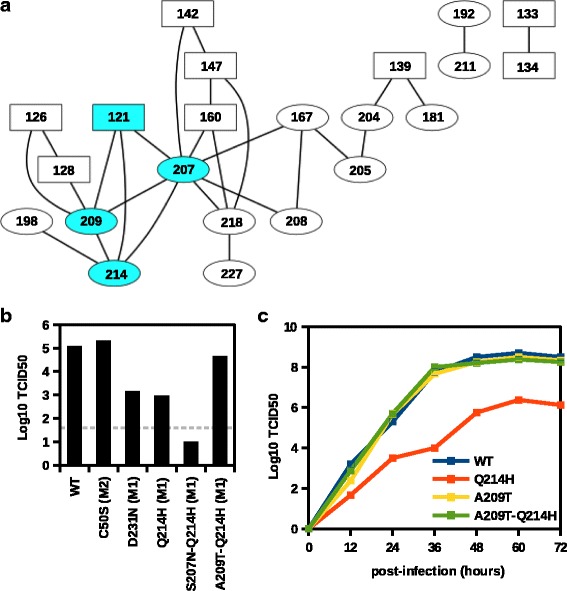
A209T as a compensatory substitution for Q214H. **a** The result from coevolution analysis on M1 protein using CAPS [50] is shown as a network. Each node represents a residue and is labeled with the amino acid position. Nodes representing residue on N-terminal domain (residues 1–164) are in rectangular shape. Nodes representing residue on C-terminal domain (residues 165–252) are in eclipse shape. An edge is drawn between coevolving residues. Residues 121, 207, 209, and 214 were identified as a coevolving group by CAPS [50] and are highlighted in cyan. **b** The TCID_50_ measured from the virus rescue experiment for the wild-type (WT) or the indicated mutant is shown. This data represent the mean value from three independent replicates. The grey dashed line represents the lower detection limit. **c** A multicycle replication assay was performed. A549 cells were infected with wild-type (WT) or the indicated mutant at an MOI of 0.005. Virus was harvested at the indicated timepoints and the TCID_50_ was measured

We were mostly interested in identifying and studying those deleterious substitutions that were prevalent in nature, if any. We then compared the RF index _*max*_ and the natural occurrence frequency for individual substitutions. This comparison was done separately for H1N1 seasonal influenza viruses (seasonal flu) and 2009 H1N1 pandemic swine influenza viruses (swine flu) using the sequence information retrieved from Influenza Research Database [47]. Interestingly, we identified three substitutions that appeared as deleterious in our high-throughput genetics data (RF index _*max*_< 0.167), yet were prevalence in naturally occurring influenza sequences (natural occurrence frequency > 50 %) (Fig. 5). These three substitutions were C50S on M2 (RF index _*max*_=0.05), D231N on M1 (RF index _*max*_=0.15), and Q214H on M1 (RF index _*max*_=0.16). These three substitutions were individually reconstructed. The deleterious effects of M1 Q214H and M1 D231N were validated by virus rescue experiment (Fig. 6b). In fact, the deleterious effect of M1 D231N was also previously demonstrated in another genetic background [48]. However, M2 C50S, which was shown to be a non-essential palmitoylation site [49], had no fitness cost in the virus rescue experiment (Fig. 6b). We postulated that either C50S was a false positive from the identification of deleterious mutations or with a fitness cost that could only be detectable under a competitive growth environment which resembled that of the high-throughput genetics experiment. Consequently, M2 C50S was ignored in the downstream analysis.

### Identification of potential compensatory substitutions by coevolution analysis

Next, we aimed to investigate the genetic mechanism of the prevalence of those deleterious substitutions in nature. One possibility was that the fitness effects of those substitutions were genetic background-dependent. In other words, substitutions which appeared as deleterious in strain A/WSN/33, the strain employed in this study, may have no fitness cost in other virus strains. We hypothesized that compensatory substitutions for those deleterious substitutions may exist in certain naturally occurring strains. Those compensatory substitutions, if they exist, could potentially be identified using phylogenetic information.

Subsequently, a coevolution analysis using CAPS [50] was performed to search for intra-protein coevolving residues (Fig. 6a). CAPS was featured by its ability to eliminate background correlations and minimize stochastic dependencies between sites using phylogenetic information. Thus, it possessed a lower false positive rate and a higher sensitivity as compared to other algorithms for detecting coevolving residues [51]. Here, CAPS was able to identified four residues (residues 121, 198, 207 and 209) on M1 that were coevolving with residue 214. In addition, CAPS detected that residues 121, 207, 209, and 214 were coevolved as a group. Residues 207 and 209 were located on the structurally unresolved M1 C-terminal domain (amino acid residues 165–252) along with residue 214, while residue 121 was located on M1 N-terminal domain (amino acid residues 1–164). Nonetheless, no residue was found to coevolve with residue 231 on M1. As a result, our analysis below focused on residue 214 and the two coevolving residues 207 and 209 that were located in the same protein domain. A significant difference in amino acid usage at these sites was detected between seasonal flu and swine flu. For seasonal flu, glutamine [Q] dominated at residue 214 (99 %), serine [S] dominated at residue 207 (93 %), and alanine [A] dominated at residue 209 (98 %). For swine flu, histidine [H] dominated at residue 214 (98 %), threonine [T] dominated at residue 209 (99 %), and asparagine [N] dominated at residue 207 (99 %). Therefore, we hypothesized that the replication defect of Q214H could be compensated by either S207N or A209T, or both of them.

We also examined the natural variant at residue 198, which was also located in the C-terminal domain and shown to be coevolving with residue 214 (Fig. 6a). Nonetheless, glutamine [Q] was dominated at residue 198 (99 %) regardless of whether the amino acid at residue 214 was glutamine [Q] or histidine [H]. It suggests that, at least in natural evolution, mutation at residue 198 was unlikely to impose a significant compensatory effect on the fitness cost Q214H.

### A209T is a compensatory substitution for Q214H

To test our hypothesis, the fitness effects of S207N and A209T on Q214H were tested by virus rescue experiment. While the addition of S207N further decreased the viral titer, addition of A209T fully restored the viral titer to WT level (Fig. 6b). A multicycle replication assay was also performed. The viral titer of Q214H was ∼100-fold lower than WT across different time points (Fig. 6c). This defect was rescued with the addition of A209T. However, A209T alone did not improve the replication kinetics above the wild type. Together, these results showed that A209T could act as a compensatory substitution for Q214H. In fact, A209T and Q214H were both located at a putative *α*-helix, helix 12 (amino acid residues 197–218), of the M1 C-terminal domain [52, 53]. It has been shown that residue 209 was one of the determinants of influenza virion morphology and spreading kinetics [54], whereas residue 214 was involved in adaptation to mice [55]. In addition, most single-amino acid substitutions at their neighboring residues, namely 210, 211, 212 and 213, were shown to attenuate the viral growth [56]. Together with our results, these evidences support the functional importance of residues 209 to 214 in viral replication. We further speculate that additional epistatic interactions may be present in this region.

The interaction between A209T and Q214H in M1 demonstrates the feasibility of identifying epistatic residues through an integration of high-throughput genetics and phylogenetic information. This analytic strategy is generally applicable to any viral gene of interest, provided that the information on natural sequence variants is available.

## Discussion

High-throughput genetics has been applied to many different genes to quantify the fitness effects of a large number of single-mutations in parallel [17]. However, high-throughput genetics alone is not sufficient to identify epistatic interactions between sites. Although our recent study has successfully profile all pairwise epistatic interactions in a 56-residue protein domain [57], the mutant library complexity, hence the cost, of such approach increases polynomially with the length of the protein. Consequently, the feasibility of profiling epistasis using high-throughput genetics alone is limited to small protein domains. By combining high throughput genetics with a phylogenetically-corrected analysis of co-evolving sites in naturally occurring sequence datasets, our approach permits the identification of epistatic residues.

Here, high-throughput genetics is performed on influenza virus A/WSN/33, which is a relatively old strain. However, most part of the high-throughput genetics data obtained in this study should be applicable to more recent strains. Previous studies have shown that high-throughput genetics data obtained from strain A/WSN/33 allowed an accurate modeling of natural evolution of influenza A virus across several decades [20, 21]. Furthermore, a recent study showed that two sets of high-throughput genetics data obtained from two strains separated by more than three decades were highly correlated [58]. Therefore, we postulate that most deleterious mutations identified in this study should carry a fitness cost when they are introduced to more recent strains. Nonetheless, we also acknowledge that additional epistatic interactions may be identified if our high-throughput genetics analysis is performed on more than one strain.

While this study focuses on a single gene, our approach can potentially be applied to study intergenic epistatic interaction. The biomedical relevance of intergenic epistasis can be highlighted by human immunodeficiency virus (HIV) resistance to protease inhibitor, in which substitutions on gag can compensate the deleterious effect associated with the drug resistance substitutions on protease [59, 60]. In fact, coevolution analysis is a major bioinformatics approach to predict protein-protein interaction [13]. We propose that by coupling with coevolution analysis of an appropriate sequence dataset, high-throughput genetics can be applied to any given interacting protein pair to search for interacting residues. Nevertheless, we do acknowledged that correlated evolution between proteins can be dominated by similar constraints on evolutionary rate but not coevolution per se [61]. Therefore, adapting our method to search for intergenic epistasis may be more challenging than to identify intragenic epistasis as described in this study.

Compensatory mutation is a type of sign epistasis [62]. In the presence of sign epistasis, the fitness effect of a given mutation could exhibit different sign (beneficial, deleterious, or neutral) depending on the genetic background. On the other hand, for magnitude epistasis, the fitness effect of a given mutation would not change sign, but would display a different magnitude depending on genetic background. Although our approach is able to identify sign epistasis, it may be difficult to adapt our approach to search for magnitude epistasis, which has a more subtle impact in fitness effect. Consequently, identification of magnitude epistasis would require a more accurate quantification of mutational fitness effects and a more sophisticated analysis to infer mutational fitness effect using phylogenetic information.

Recently, there is an increasing interest in higher-order epistasis, which describes the epistatic interaction between more than two mutations [63]. While this study focuses on pairwise epistasis, we propose that our approach can be adapted to search for higher-order epistasis. For example, higher-order epistasis can potentially be identified by deleterious mutations that emerged as a group in natural evolution, where each mutation within the group alone is deleterious but the entire group of mutations together has a neutral or beneficial fitness effect. Therefore, combining phylogenetic information and high-throughput genetics can potentially facilitate the understanding of higher-order epistasis in natural evolution.

During the course of our work, Melamed et al. published a study that integrated high-throughput genetics with multiple sequence alignment of evolutionarily divergent variants to identify protein-binding sites on *Saccharomyces cerevisiae* poly(A)-binding protein, Pab1 [64]. More specifically, they have demonstrated that deleterious substitutions that naturally existed could be due to the evolutionary divergence of functional interface. While their aim and approach are different from our work here, both Melamed et al. and this study suggest that high-throughput genetics and natural sequence variation can be synergistic for mapping protein sequence-function relationship.

Our recent study has indicated that functional residues can be efficiently identified by combining protein structure information and high-throughput genetics [23]. In this study, protein structure information was not extensively utilized due to the absence of structural information in the region of interest (M1 C-terminal domain). Nevertheless, it is shown that combining coevolution analysis with structural information improves the identification of residue interactions [65], and helps classify the type of coevolution (functional versus structural coevolution) [50, 66]. Therefore, protein structure information can be highly valuable for mapping epistatic interaction. Future approach for studying second or higher-order interactions may integrate phylogenetic information, protein structure information and high-throughput genetics.

## Conclusions

This work demonstrates a hybrid strategy to identify epistatic residues by combining phylogenetic information and high-throughput genetics. We successfully identified the epistatic interaction between influenza A virus M1 substitutions A209T and Q214H. While our proof-of-concept is based on a viral protein, our approach can potentially be applied to probe for epistatic residues in any protein of interest, provided that the phylogenetic information is available.

## Methods

### Viral mutant library and point mutations

In this study, M segment of influenza virus was analyzed by high-throughput genetics. To increase the statistical confidence in the fitness profiling result, two different mutant library building strategies were employed in this study, namely the whole segment mutant library and the “small libraries”. The methodologies for construction of these two libraries using error-prone PCR were described in our previously studies [19, 23]. For the whole segment mutant library, the entire M segment was subjected to mutagenesis. The M segment mutant library plasmids for both the whole segment mutant library or the “small libraries” were created by performing error-prone PCR on the M segment of the eight-plasmid reverse genetics system of influenza A/WSN/1933 (H1N1) [67]. Mutated insert was generated by PCR using error-prone polymerase Mutazyme II (Stratagene, La Jolla, CA) with the following primers:

■■■ Whole segment library insert: 5’-GTG TGT CGT CTC GGG AGC AAA AGC AGG TAG ATA TTG AAA GAT G-3’ and 5’-GTG TGT CGT CTC GTA TTA GTA GAA ACA AGG TAG TTT TTT ACT CC-3’
■■■ Small library 1 insert: 5’-AAG CAG CGT CTC ATT GAA AGA TGA GTC TTC TAA CC-3’ and 5’-AAC TGC CGT CTC AAT GTT ATT TGG ATC TCC GTT CCC-3’
■■■ Small library 2 insert: 5’-CAC GTC TCA GCT TTG TCC AAA ATG CTC TTA AT-3’ and 5’-CAC GTC TCA TTA GTG GAT TGG TTG TTG TCA C-3’
■■■ Small library 3 insert: 5’-CAC GTC TCA GCA TCG GTC TCA TAG GCA AAT G-3’ and 5’-CAC GTC TCA ACT TGA ATC GTT GCA TCT GCA C-3’
■■■ Small library 4 insert: 5’-CAC GTC TCA GAT GAT CTT CTT GAA AAT TTA CAG-3’ and 5’-CAC GTC TCA CAG CTC TAT GTT GAC AAA ATG A-3’

The BsmBI-digested pHW2000 plasmid [67] was used as the vector for the whole segment mutant library, whereas the corresponding vector for each of the three “small libraries” was generated by PCR with KOD DNA polymerase (EMD Millipore, Billerica, MA) using the following primers:

■■■ Small library 1 vector: 5’-CAC GTC TCA TCA ATA TCT ACC TGC TTT TGC TC-3’ and 5’-CAC GTC TCA ACA TGG ACA AAG CAG TTA AAC TG-3’
■■■ Small library 2 vector: 5’-CAC GTC TCA AAG CGT CTA CGC TGC AGT CCC-3’ and 5’-CAC GTC TCA CTA ATC AGA CAT GAG AAC AGA AT-3’
■■■ Small library 3 vector: 5’-CAC GTC TCA ATG CTG GGA GTC AGC AAT CTG TT-3’ and 5’-CAC GTC TCA AAG TGA TCC TCT CGT CAT TGC AG-3’
■■■ Small library 4 vector: 5’-CAA CGT CTC ACA TCT TTT AGA CCA GCA CTG GAG CTA G-3’ and 5’-TTG TCA CGT CTC AGC TGG AGT AAA AAA CTA CCT TG-3’

Both the insert and the vector were then digested by BsmBI (New England Biolabs, Ipswich, MA). For each mutant library, The corresponding insert and vector were ligated using T4 DNA ligase (Life Technologies, Carlsbad, CA), and transformed into electrocompetent MegaX DH10B T1R cells (Life Technologies). Subsequently, ∼200,000 colonies were scraped and directly processed for plasmid DNA purification (Qiagen Sciences, Germantown, MD). Point mutations for the validation experiment were constructed using the QuikChange XL Mutagenesis kit (Stratagene) according to the manufacturer’s instructions.

The whole segment mutant library and the “small libraries” had their own pros and cons associated with the deep sequencing strategy. Illumina MiSeq 2 × 250 bp sequencing was employed in the “small libraries” approach. Since each sequencing read covered the entire mutagenized region, the haplotype for a given clone could be examined. Therefore, fitness effects arouse from mutation interactions could be filtered in the “small libraries” approach. In contrast, genetic linkage between mutations could not be addressed in the whole segment library due to the long span of the mutagenized region. Thus, fitness effects arouse from mutation interactions cannot be precisely accounted for. However, Illumina HiSeq 2000 2 × 100 bp sequencing was employed in the whole segment mutant library approach, which offered a much deeper coverage to increase confident in computing fitness effect. Therefore, the profiling results from these two different strategies would complement each other.

### Transfections, infections, and titering

293T cells (human embryonic kidney cells) were transfected with Lipofectamine 2000 (Life Technologies) using the M segment mutant library plasmid (for screening purpose) or point mutation plasmid (for validation purpose) plus 7 other wild-type plasmids. Supernatant was replaced with fresh cell growth medium at 24-hour and 48-hour post-transfection. At 72-hour post-transfection, supernatant containing infectious virus was harvested, filtered through a 0.45 um MCE filter, and stored at −80 °C. The viral titer (concentration of infectious particles) was measured by 50 % Tissue Culture Infective Dose (TCID_50_) using on A549 cells (human lung carcinoma cells). In this study, ∼5 million 293T cells were employed for transfection of each mutant library. We believed this amount of 293T cells were not sufficient to reconstitute all genotypes and would create a huge bottleneck in genetic diversity. If ∼35 million 293T cells (7-fold increase in cell number) were used instead as indicated in our recent study [23], the bottleneck at the transfection step could be hugely relieved and the correlation of RF indices between replicates would be greatly improved.

Virus produced from the 293T transfection was used to infect A549 cells at a multiplicity of infection (MOI) of 0.05. MOI represented the infectious virus to cell ratio. Infected cells were washed with PBS followed by the addition of fresh cell growth medium at 2-hour post-infection. Virus was harvested at 24-hour post-infection for screening experiment and validation, or at indicated time point for growth curve experiment.

### Sequencing library preparation

Viral RNA was extracted from the post-infection viral mutant library using QIAamp Viral RNA Mini Kit (Qiagen Sciences) and was reverse transcribed to cDNA using Superscript III reverse transcriptase (Life Technologies).

For the whole segment mutant library, DNA from the plasmid library or cDNA from the post-infection viral mutant library were amplified using the following primers:

■■■ Amplicon 1: 5’-CTA CAC GAC GCT CTT CCG ATC TNN NNN NAG ATG AGT CTT CTA ACC GAG-3’ and 5’-TGC TGA ACC GCT CTT CCG ATC TNN NNN NCC TAA AAT CCC CTT AGT CAG-3’
■■■ Amplicon 2: 5’-CTA CAC GAC GCT CTT CCG ATC TNN NNN NAA GAC CAA TCC TGT CAC CT-3’ and 5’-TGC TGA ACC GCT CTT CCG ATC TNN NNN NGA ATG TTA TCT CCC TCT TAA G-3’
■■■ Amplicon 3: 5’-CTA CAC GAC GCT CTT CCG ATC TNN NNN NGC AGT TAA ACT GTA TAG GAA G-3’ and 5’-TGC TGA ACC GCT CTT CCG ATC TNN NNN NAG TCA GCA ATC TGT TCA CAG-3’
■■■ Amplicon 4: 5’-CTA CAC GAC GCT CTT CCG ATC TNN NNN NTG GCC TGG TAT GCG CAA C-3’ and 5’-TGC TGA ACC GCT CTT CCG ATC TNN NNN NAA TAT CCA TGG CCT CTG CT-3’
■■■ Amplicon 5: 5’-CTA CAC GAC GCT CTT CCG ATC TNN NNN NTG GAT CGA GTG AGC AAG C-3’ and 5’-TGC TGA ACC GCT CTT CCG ATC TNN NNN NGG ATC ACT TGA ATC GTT GC-3’
■■■ Amplicon 6: 5’-CTA CAC GAC GCT CTT CCG ATC TNN NNN NAA CGA ATG GGG GTG CAG AT-3’ and 5’-TGC TGA ACC GCT CTT CCG ATC TNN NNN NCC CTC ATA GAC TCT GGC A-3’
■■■ Amplicon 7: 5’-CTA CAC GAC GCT CTT CCG ATC TNN NNN NAC TTG ATA TTG TGG ATT CTT GA-3’ and 5’-TGC TGA ACC GCT CTT CCG ATC TNN NNN NTA CTC CAG CTC TAT GTT GAC-3’

Following PCR, 7 amplicon products were pooled together. 0.875 million copies of the pooled product were used as the input for the second PCR, which was equivalent to 10 paired-end reads per molecule if 8.75 million paired-end reads were sequenced. 5’-AAT GAT ACG GCG ACC ACC GAG ATC TA CAC TCT TTC CCT ACA CGA CGC TCT TCC G-3’ and 5’-CAA GCA GAA GAC GGC ATA CGA GAT CGG TCT CGG CAT TCC TGC TGA ACC GCT CTT CCG-3’ were used as the primers for the second PCR. Products of the second PCR were submitted for deep sequencing using Illumina HiSeq 2000 with 100 bp paired-end reads.

For the “small libraries”, DNA from the plasmid library or cDNA from the post-infection viral mutant library were amplified using the following primers:

■■■ Small library 1: 5’-TAG ATA CTG GAG GAT GAG TCT TCT AAC C-3’ and 5’-TGT CCA CTG GAG TTG GAT CTC CGT TCC C-3’
■■■ Small library 2: 5’-TAG ACG CTG GAG CCA AAA TGC TCT TAA T-3’ and 5’-GTC TGA CTG GAG GAT TGG TTG TTG TCA C-3’
■■■ Small library 3: 5’-CTC CCA CTG GAG GTC TCA TAG GCA AAT G-3’ and 5’-AGG ATC CTG GAG ATC GTT GCA TCT GCA C-3’
■■■ Small library 4: 5’-AAA AGA CTG GAG TCT TGA AAA TTT ACA G-3’ and 5’-TTA CTC CTG GAG TAT GTT GAC AAA ATG A-3’

The resulting PCR amplicons were digested with BpmI (New England Biolabs), end-repaired by end repair module (New England BioLabs), and 3’ dA-tailed by dA-tailing module (New England BioLabs). dA-tailed amplicons were ligated to sequencing adapters using T4 DNA ligase (Life Technologies) as previously described [23]. The adapter-ligated products were enriched by a final PCR using primers: 5’-AAT GAT ACG GCG ACC ACC GAG ATC TAC ACT CTT TCC CTA CAC GAC-3’ and 5’-CAA GCA GAA GAC GGC ATA CGA GAT CGG TCT CGG CAT TCC TGC TGA ACC-3’. Deep sequencing was performed using Illumina MiSeq with 250 bp paired-end reads. Raw sequencing data have been submitted to the NIH Short Read Archive under accession number: BioProject PRJNA285135.

### Data analysis

Sequencing data were processed as described previously for whole segment library [19] and for the “small libraries” [23]. To increase statistic confidence in computing RF index, two filters were applied as follow. 1) Those mutations with an input count of < 30 error-corrected reads in the whole segment mutant library were discarded. 2) All C to A and G to T mutations were discarded due to an observed DNA oxidative damage in sequencing library preparation [68].

We aimed to identify deleterious mutations with high confidence. Applying high-throughput genetics using the influenza virus eight-plasmid reverse genetic system [67] could produce many false positives in identifying deleterious mutations − a significant number of neutral mutations may display as deleterious in the fitness profiling result. This caveat was largely due to the huge bottleneck effect in the transfection step, which was observed in multiple studies [19–21]. Briefly, each independently transfected viral mutant library was an incomplete sampling of mutants in the plasmid mutant library. To minimize the artifact brought by the bottleneck effect, a conservative estimate would be needed to compute the fitness effect of individual point mutations for the purpose of identifying deleterious mutations. As a result, for each mutation, the RF index _*max*_, which represented the highest value among the five RF indices from five biological replicates, was used for the downstream analysis unless otherwise stated. The RF indices are listed in Additional file 1. Those mutations in the “small libraries” with an input frequency of < 10-fold of the baseline frequency are listed as “NA”. Baseline frequency represented the mutation introduced in sequencing library preparation and was determined by sequencing the WT plasmid.

True positive rate (TPR) was computed by:

$${\fontsize{9.4pt}{9.3pt}{}\begin{aligned} \text{TPR} = \frac{\text{\textit{number of nonsense mutations below fitness cutof f}}}{\text{\textit{number of all nonsense mutations}}} \end{aligned}} $$

False positive rate (FPR) was computed by:

$${}\begin{aligned} \text{FPR} = \frac{\text{\textit{number of silent mutations below fitness cutof f}}}{\text{\textit{number of all silent mutations}}} \end{aligned} $$

Mutations resided within 200 bp from the termini of the M segment were not considered in computing TPR and FPR since mutations at the terminus regions could impose a fitness cost by interrupting the *cis*-acting packaging signal [69], which was independent of the change in amino acid sequence.

### Structural analysis

DSSP (http://www.cmbi.ru.nl/dssp.html) was used to compute the solvent accessible surface area (SASA) for each residue from the PDB structure [70]. SASA was then normalized to the empirical scale reported in [71] to obtain relative solvent accessibility (RSA). RSA was computed for all except the terminal residues of both chain A and chain B of the M1 dimer in both dimeric form and monomeric form (PDB: 1EA3) [39]. Residues with an RSA greater than 0.2 in both monomeric chain A and monomeric chain B were classified as surface-exposed residues (“Exposed” in Fig. 4d), as buried residues otherwise (“Buried” in Fig. 4d). For each residue, the ratio between the RSA computed from the dimeric form to the RSA computed from the monomeric form was calculated, and was notated by RSA _*dimeric*_/RSA _*monomeric*_. This ratio represented the reduction of RSA during M1 dimerization and was always less than or equal to 1. Residue that was extensively involved in the dimeric interface would have a low RSA _*dimeric*_/RSA _*monomeric*_. Here, we defined those surface-exposed residues that had a RSA _*dimeric*_/RSA _*monomeric*_ of less than 0.5 as dimer-interface residues (“Interface” in Fig. 4d).

### Coevolution analysis

Protein sequences were obtained from Influenza Research Database (www.fludb.org) [47] on August 29, 2014. The sequence searching criteria included complete M1 or M2 protein sequences of human influenza A virus H1N1 subtype from all geographical locations with duplicate sequences removed. Additionally, the option of “Exclude all pH1N1 proteins” was applied to obtain the protein sequence information of the seasonal influenza virus strains, and the option of “Include only pH1N1 proteins” was applied to obtain the protein sequence information of the 2009 H1N1 pandemic swine influenza virus strains. The sampling dates ranged from 1918 to 2014. One protein sequence with a different length as compared to A/WSN/33 was removed. Subsequently, a total of 150 sequences of the seasonal influenza virus strains and 278 sequences of the 2009 pandemic swine influenza virus strains were included in the downstream analysis. CAPS was employed for identification of coevolving residues [50]. All 428 sequences obtained from Influenza Research Database were used for coevolution analysis with default parameters. Of note, this set of sequences did not contain any laboratory strain.

## Additional file

Additional file 1
**Supplementary Dataset 1: RF index for each mutation.** (XLS 364 kb)
